# Deep Learning-Based Segmentation and Quantification of Retinal Capillary Non-Perfusion on Ultra-Wide-Field Retinal Fluorescein Angiography

**DOI:** 10.3390/jcm9082537

**Published:** 2020-08-06

**Authors:** Joan M. Nunez do Rio, Piyali Sen, Rajna Rasheed, Akanksha Bagchi, Luke Nicholson, Adam M. Dubis, Christos Bergeles, Sobha Sivaprasad

**Affiliations:** 1 Institute of Ophthalmology, University College London, London EC1V 9EL, UK; piyali.sen.18@ucl.ac.uk (P.S.); rasheed.rajna@nhs.net (R.R.); luke.nicholson3@nhs.net (L.N.); a.dubis@ucl.ac.uk (A.M.D.); sobha.sivaprasad@nhs.net (S.S.); 2 NIHR Moorfields Biomedical Research Centre, Moorfields Eye Hospital, London EC1V 2PD, UK; akanksha.bagchi@gmail.com; 3 King’s College London, School of Biomedical Engineering & Imaging Sciences, London SE1 7EU, UK

**Keywords:** retinal non-perfusion, fluorescein angiography, image segmentation

## Abstract

Reliable outcome measures are required for clinical trials investigating novel agents for preventing progression of capillary non-perfusion (CNP) in retinal vascular diseases. Currently, accurate quantification of topographical distribution of CNP on ultrawide field fluorescein angiography (UWF-FA) by retinal experts is subjective and lack standardisation. A U-net style network was trained to extract a dense segmentation of CNP from a newly created dataset of 75 UWF-FA images. A subset of 20 images was also segmented by a second expert grader for inter-grader reliability evaluation. Further, a circular grid centred on the FAZ was used to provide standardised CNP distribution analysis. The model for dense segmentation was five-fold cross-validated achieving area under the receiving operating characteristic of 0.82 (0.03) and area under precision-recall curve 0.73 (0.05). Inter-grader assessment on the 20 image subset achieves: precision 59.34 (10.92), recall 76.99 (12.5), and dice similarity coefficient (DSC) 65.51 (4.91), and the centred operating point of the automated model reached: precision 64.41 (13.66), recall 70.02 (16.2), and DSC 66.09 (13.32). Agreement of CNP grid assessment reached: Kappa 0.55 (0.03), perfused intraclass correlation (ICC) 0.89 (0.77, 0.93), non-perfused ICC 0.86 (0.73, 0.92), inter-grader agreement of CNP grid assessment values are Kappa 0.43 (0.03), perfused ICC 0.70 (0.48, 0.83), non-perfused ICC 0.71 (0.48, 0.83). Automated dense segmentation of CNP in UWF-FA images achieves performance levels comparable to inter-grader agreement values. A grid placed on the deep learning-based automatic segmentation of CNP generates a reliable and quantifiable method of measurement of CNP, to overcome the subjectivity of human graders.

## 1. Introduction

Retinal vascular diseases are common causes of visual impairment [1]. A major cause of visual morbidity is retinal capillary non-perfusion (CNP), as it triggers the release of vascular endothelial growth factor, which in turn results in macular oedema and retinal neovascularisation. Fluorescein angiography (FA) is the standard tool used for diagnosing CNP. Areas of CNP are identified as a loss of normal texture of the greyscale of the background fluorescence, that may range from a mild loss of greyness to a complete dark area of hypofluorescence, based on the density of drop out of the superficial capillary plexus [2]. Accurate identification of CNP is challenging due to the subjectivity of assessing the gradations of the grayscale. In addition, CNP area may range from single or multiple small areas to large confluent regions and the manual quantification of CNP is time-consuming and not practical in clinical practice. With the advent of ultra-wide field (UWF) imaging system, FA done on UWF (UWF-FA) has become the standard diagnostic tool for understanding the extent of CNP area, despite the fact that grading far periphery for CNP remains challenging. 

Although some of the constraints of FA in assessing CNP were resolved by the recent introduction of optical coherence tomography angiography (OCT-A), these images do not provide information on associated retinal leakage, and artefacts remain a challenge. Wide-angled OCT-A is still in its infancy, costly, not widely used, and is riddled with artefacts, as the retina is scanned from central retina to periphery. Additionally, we have over fifty years of knowledge on FA that we do not have with OCT-A. 

With the advent of new investigational products aimed to prevent or reverse CNP, it is now useful to quantify CNP topographically as an end-point in clinical trials, and for routine clinical assessment. We have developed a reliable concentric ring method to quantify the area of CNP, by dividing the gradable retina into 72 segments (6 rings, each divided into 12 segments) [3]. This methodology allows one to record CNP location by graders labelling each segment as perfused or non-perfused. However, we remain reliant on graders to grade the perfused and non-perfused areas, decreasing the reliability of quantification of CNP. 

Deep learning-based tools have showcased a range of potential benefits, and a wealth of challenges in assessing retinal diseases [4,5,6,7]. Nevertheless, the accurate assessment and location of CNP areas has attracted less attention. Jasiobedzki et al. reported an algorithm to detect non-perfused regions on FA images, based on mathematical morphology [8]. The image was initially over-segmented in primary regions, where texture, as a measure of the size and number of capillaries, was analysed. The authors reported agreement with an ophthalmologist in a single image. Buchanan et al. presented a method to locate ischemic regions in Optos UWF-FA images based on contextual knowledge [9]. Multiple classifiers were implemented to detect different pathology signs (distance from vessel bifurcations, density of vessels, presence of leakages and microaneyrysms), and collected evidence was combined by an AdaBoost algorithm. The authors used 16 UWF-FA sequences (9 ischaemic) of 15 to 60 frames. Other methods relied on modelling CNP regions as intensity valleys [10,11]. Their performance strongly depends on illumination correction techniques to account for non-uniformities, homomorphic filtering and camera-model based solutions. Zheng et al. proposed a texture segmentation framework which combined an unsupervised step to identify candidate regions and a supervised weighted AdaBoost classifier [12]. The framework was separately trained and validated on 40 malarial retinopathy patients, and reported 73.0% sensitivity and 90.8% specificity for best parameters and 10 FA images from diabetic retinopathy patients reporting 72.6% sensitivity, 82.1% and specificity for best parameters.

The aim of our study was to develop a reliable methodology to quantify CNP topographically in UWF-FA images, so that subjectivity induced by human graders can be avoided (Source code available at https://github.com/RViMLab/JCM2020-Non-Perfusion-Segmentation). The methodology involved two stages. First, we used a fully convolutional deep learning method to provide CNP dense segmentation. Then, the concentric grid method was used to quantify the topographical distribution.

## 2. Materials and Methods

### 2.1. Dataset

This secondary analysis of anonymised FA images captured on UWF was performed from January 2017 to March 2020. The images were obtained from two multicentre randomised clinical trials on diabetic retinopathy [13,14]. Ethical approval was obtained to use these images from clinical trials without further informed consent for this study. Institutional review board approval was obtained from the London-Westminster Research Ethics Committee for analysis of anonymised data, and the study was conducted in accordance with the tenets of the Declaration of Helsinki.

All images were captured by an Optos 200Tx (Optos, Plc, Dumferline, Scotland). UWF-FA images were attained using a standard protocol after intravenous bolus infusion of 5 mL of 20% fluorescein sodium. The protocol used in the clinical trials mirrors the procedure used in clinical practice and consisted of acquiring images in the transit phase (up to 20 s), arteriovenous phase (up to 45 s–1 min), and late frames, at 3–4 min and 7–8 min.

Researchers can apply to Moorfields Research Management Committee for access to the data for use in an ethics approved project on moorfields.resadmin@nhs.net.

### 2.2. Curation of Dataset

Two investigators, P.S. and A.B., curated a 75-representative FA image dataset encompassing different degrees and characteristics of retinas affected by CNP, due to diabetic retinopathy. Images in the early arteriovenous phase were selected, ranging approximately between 25 and 55 s of transit time since dye injection, to allow the images to reach the peak phase of maximal fluorescence, while precluding the appearance of staining and leakage that would hinder the visualization of CNP regions.

The informative area of the image was defined as the gradable retinal region. There, all anatomical structures were sufficiently visible alongside the regions of CNP on the selected angiogram frame. Non-informative regions, usually found in the periphery, present a contrast that is insufficient to visualise CNP with an acceptable certainty.

**Figure 1:**
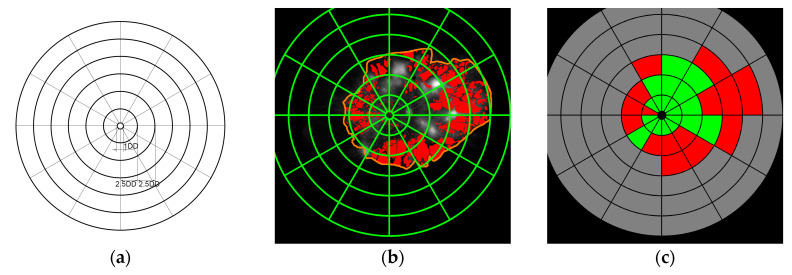
Circular grid assessment of capillary non-perfusion (CNP) dense segmentation. (**a**) Circular grid template; (**b**) Grid on dense segmentation for automatic assessment (perfusion (red), gradable retina (orange)); (**c**) Automated grid assessment (perfused (green), non-perfused (red), ungradable (grey).

One expert grader (P.S.) with medical retina fellowship manually demarcated the outer boundary of the gradable retina. Within its boundary, CNP regions were manually segmented, and pixel-level masks were created (Figure 1). Areas within the gradable retina were considered to be perfused if they showed the normal ground-glass appearance on FA. Contrary, areas of CNP were defined as hypofluorescent areas of lesser grayscale than the normal ground-glass appearance associated with the absence of retinal arterioles and/or capillaries and pruned appearance of adjacent arteriole [15]. To assess inter-grader variability, CNP was also segmented in 20 images by a second retinal fellowship trained grader (R.R.).

### 2.3. Automated CNP Segmentation

#### 2.3.1. Pre-Processing

Each image was pre-processed to decrease computational demand and ease network convergence by resizing the central 2000 × 2000 region to 448 × 448, and normalizing pixel values to 0.5 mean and 1.0 standard deviation. To augment the training set and tackle deep learning networks’ tendency to overfit on training data, resized cropped images underwent random contrast shift ((μ+γ(x−μ), x was the original value, μ was the average contrast, γ was a factor between 0.8 and 1.2) and brightness shift (scaling by a random positive factor <0.1), horizontal and vertical flipping, and smooth shear deformations (defined by random values of rotation angle and shearing factors between (−0.25, 0.25) to achieve physiologically plausible images). The resulting images were cropped to a patch keeping over 63% of their area to increase data, while keeping most of the gradable region (random axis ratios of up to 0.7). All random values were extracted from a uniform distribution. Bilinear interpolation was used in any transformation requiring resampling.

#### 2.3.2. Network

The developed network’s architecture follows the U-net symmetric encoding-decoding structure [16]. The network combines a downsampling path that extracts spatial features from the input image, and an upsampling path to construct the dense segmentation map. The downsampling path consists of the repeated application of blocks, including two 3 × 3 convolutions and a max pooling operation layer, with a pooling size of 2 × 2 and stride of 2. This sequence is repeated four times, doubling the number of feature channels at each step, and connected to the upsampling path by blocks, including two 3 × 3 convolution operations. The upsampling path also consists of four repetitions of identical blocks, including an upsampling operation. A 1 × 1 convolution is added after the final upsampling block, to generate the desired number of classes from the last 64-channel feature map.

**Figure 2:**
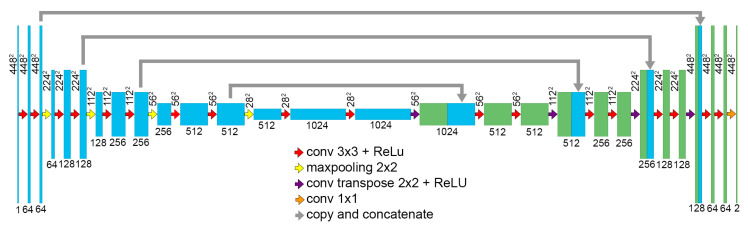
Segmentation network.

The downsampling and upsampling paths are connected by similar blocks including two 3 × 3 convolutions and a drop-out layer [17]. Moreover, the corresponding downsampling and upsampling blocks are connected by skip connections. The feature maps resulting from the second convolution in the upsampling blocks are concatenated to the feature maps resulting from the upsampling operation in the corresponding upsampling block (Figure 2).

#### 2.3.3. Training Methodology

The network was trained using a Tensorflow distributed machine learning system [18]. Stochastic gradient descent was used to update the parameters and batch size 1, to contribute to regularization. The networks at each fold were trained for 33K epochs to prevent overfitting, starting with a learning rate of 0.05, with 0.9 decay rate every 1000 epochs, in conjunction with a momentum of 0.9 [19]. The dropout ratio was set to 0.5. The experiments were performed on a NVidia Quadro P6000 GPU. Training was carried out with the goal of optimising the inverse of generalized dice coefficient as the objective function, defined at pixel level as:

(1) $$GDL=\;1-2\frac{\sum_{l=1}^{2}w_{l}\sum_{n}r_{ln}p_{ln}}{\sum_{l=1}^{2}w_{l}\sum_{n}r_{ln}+p_{ln}},$$

where $r_{n}$ are the reference pixel values, $p_{n}$ are the probabilistic predicted values and $w_{l}=1/{\left(\overset{N}{\underset{1}{\sum }}r_{ln}\right)}^{2}$ provides invariance to imbalanced label set distributions, by correcting the contribution of each label, l, by the inverse of its size [20].

### 2.4. Quantification of Capillary Non-Perfusion

We used a validated concentric rings method to quantify the topographical representation of CNP on UWF-FA [3]. The concentric ring template consists of a central circle and 6 rings, with diameters measured in disc diameters (DD). The innermost circle was centred on the fovea and had a diameter of 1 DD. The first ring, the macular ring, was 2.5 DD in radius, and each subsequent ring’s radius was incremented by 2.5 DD. All rings were divided into 12 segments subtending 30 degrees (Figure 1) [3]. The innermost circle, corresponding to the FAZ, was excluded from grading. The ring template was superimposed on each UWF-FA image, and each segment was graded as one of the following two possibilities: “perfused” (P), “non-perfused” (N), if more than 50% of the segment was considered to be within the same category. The second grader graded all images for the purpose of inter-grader agreement evaluation. These measured the subjective assessment of CNP by human graders. For the purpose of automatic assessment, the circular grid was placed on the automated segmentation, and evaluated using the same procedure. Each segment where the ratio of CNP pixels surpassed 30% was labelled as non-perfused (example in Figure 1). The threshold value was object of experimentation and established empirically. We evaluated 3 different thresholds: 30%, 40% and 50%, and observed that 30% was the best threshold where the graders tended to “see” more CNP when grading the grid.

### 2.5. Data Analysis

To test the robustness of the segmentation, the model was five-fold cross-validated, using randomly created folds from the 75 UWF-FA dataset. The performance of the different folds was evaluated using AUC (area under the receiving operating characteristic, ROC) and AUPRC (area under precision-recall curves, PCR). ROC curves illustrate the relation between the true positive rate (TPR, a.k.a. sensitivity or recall) and the false positive rate (FPR) when the discrimination threshold of the model is varied. PCR curves similarly describe the relation between precision (PR) and recall (RC). Precision (resp. recall) is the measure of effectiveness in identifying positive pixels among all pixels identified as positives (resp. all pixels labelled as positives). Thus, PRC curves are commonly suggested for class imbalanced data. Curves for the different folds were averaged, and the corresponding AUC and AUPRC were studied.

We defined the automated segmentation at the centred operating point (middle discriminative threshold) as an independent grader. Performance results were computed independently for all images in every test fold, and averaged through the whole dataset. Both dense segmentation and concentric grid assessment were evaluated, as well as inter-grader agreement. Reduced consensus regarding the gradable retina region led us to limit the assessment of inter-grader variability to CNP segmentation within the gradable retina provided by the first grader.

For quantitative analysis of the experimental results on dense segmentation, precision, recall, and dice coefficient, that (DSC) combines PR and RC notions, were studied. Using values of true positives (TP), true negatives (TN), false positives (FP) and false negatives (FN), the metrics are defined as PR=TP / (TP+FP), RC=TPR=TP / (TP+FN) and DSC=2TP / (2TP+FP+FN). 

Cohen’s kappa coefficient was used to evaluate concordance between circular grid assessments. We used Cohen’s kappa coefficient as a measure of agreement for categorical data that considers agreement by chance [21,22]. The concordance between human graders (inter-grader reliability) and automated assessments of dense segmentations (automated dense segmentation and human grander manual segmentations) were evaluated. Following standard protocols in perfusion inter-rater assessment, kappa statistics were independently evaluated for each image, and averaged through the entire dataset (standard error is also reported) [23]. Unweighted kappa values were used to equally emphasize different kinds of disagreement.

We also investigated the concordance between the average number of perfused and non-perfused segments identified by the automated methodology and grader 1. As a measure of agreement between quantitative measurements, intraclass correlation coefficients (ICC), and 95% confidence intervals (CI), were evaluated [24]. The number of perfused and non-perfused segments by both grader 1 and the automated methodology were also independently investigated for each of the six rings demarcated by the grid.

## 3. Results

### 3.1. Dense Segmentation

**Figure 3:**
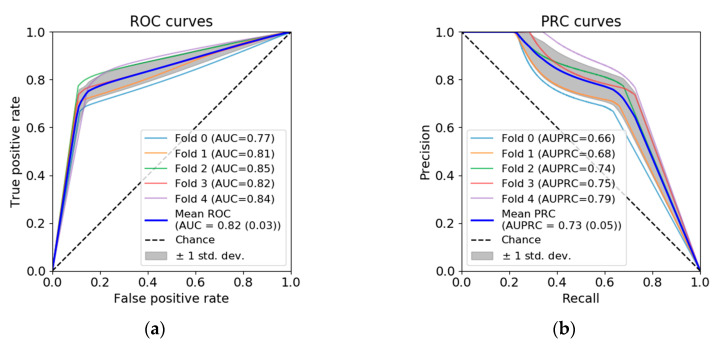
Automated dense segmentation of CNP. (**a**) ROC curve. (**b**) PRC curve.

ROC and PRC curves, and the corresponding AUC and AUPRC, are shown in Figure 3. AUC in the different folds were 0.77, 0.81, 0.85, 0.82 and 0.84. AUC for the mean ROC was 0.82 (0.03). AUPRC in the different folds were 0.66, 0.68, 0.74, 0.75 and 0.79. AUPRC for the mean PRC was 0.73 (0.05).

**Figure 4:**
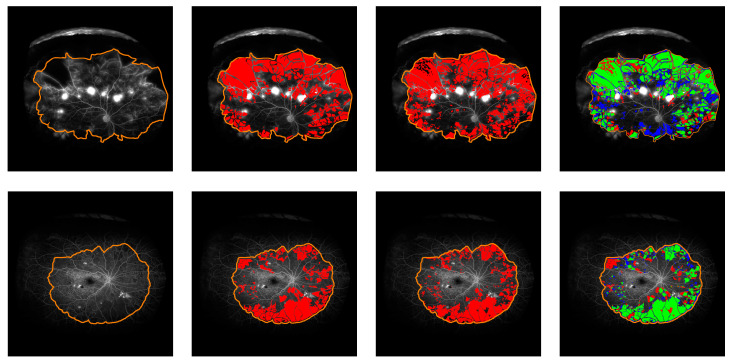
CNP dense segmentation examples. Time after dye injection: 53 (**top**) and 43 (**bottom**) seconds. Left to right: FA image and gradable area, manual segmentation, automatic segmentation, performance comparison (TP: green, FN: red, FP: blue).

Inter-grader agreement computed through the subset of 20 images reaches 59.34 (10.92) precision, 76.99 (12.5) recall, and 65.51 (4.91) DSC, with grader one annotating 25.63% (12.81) of the gradable retina as perfused, compared to a remarkably higher value of 36.33% (12.93) of perfusion annotated by grader 2 within the same gradable retina. The automated segmentation provided by the model at the centred operating point within the gradable retina shows similar values to the ones reached by the inter-grader experiment: 64.41 (13.66) precision, 70.02 (16.2) recall, and 66.09 (13.32) DSC, labelling 27.73% (13.72) of the gradable retina as non-perfused (see examples in Figure 4).

### 3.2. Quantification of CNP in the Concentric Grid

**Figure 5:**
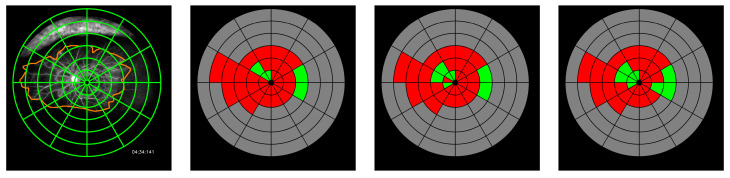

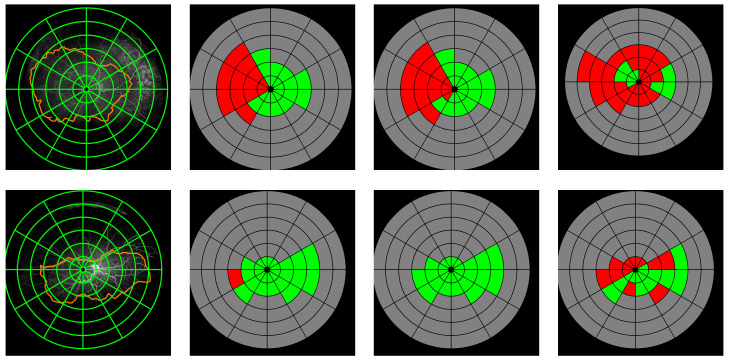
Grid assessment examples. Left to right: FA image, grid annotation from grader 1, automated assessment, grid annotation from grader 2 (Kappa values. Ex. 1: Auto 0.80, Grid2 0.65; Ex. 2: Auto 0.93 Grid2 0.74; Ex.3: Auto 0, Grid2 0.08).

Agreement between concentric grid assessments is reported in Table 1. The circular grid assessment extracted from the automatic segmentation and the manual assessment provided by the first grader show an agreement of 0.55 (0.03) kappa. ICC of perfused and non-perfused segments are 0.89 (0.77, 0.93) and 0.86 (0.73, 0.92), respectively. When the grid assessment is performed on dense CNP annotations provided by the first grader, agreement with automatic assessment reaches 0.65 (0.04) kappa. In this case, ICC values are 0.90 (0.85, 0.94) for perfused segments, and 0.88 (0.81, 0.92) for non-perfused segments. Inter-grader agreement between manual assessment of CNP by the two graders on the circular grid shows 0.43 (0.03) kappa. ICC for perfused and non-perfused segments are 0.70 (0.48, 0.83) and 0.71 (0.48, 0.83). Results on the average number of perfused and non-perfused segments in each ring are listed in Table 2. Some examples are illustrated by Figure 5.

## 4. Discussion

In our study, we explored the viability of deep learning-based quantification of CNP, and propose an implementation that demonstrates this technology can be used to diagnose and monitor CNP progression. Our implementation uses a fully convolutional deep learning method to extract a CNP dense segmentation. Subsequently, an automatic grid assessment is performed, to provide a repeatable description of the topographical distribution of the CNP regions.

Developing and validating deep learning algorithms is a rapidly advancing field in medicine, with a desire to rapidly translate to practice, to ease the pressures of the exponential increase in clinic appointments in all health sectors. Whilst most of these algorithms are developed to diagnose conditions that can be already well-ascertained by clinicians, they also have a role in situations where clinicians face challenges in accurately measuring pathological findings, even if time factor is not an issue. Here, we report a cautious approach of development and validation of a supervised deep learning algorithm in such a scenario, and enumerate the challenges faced and the proposed solutions. The study is performed on a newly created dataset limited to 75 UWF-FA images, manually segmented and graded by experts. Please note that the dataset’s size compares favorably with complementary datasets (DRIVE, STARE) used for assessing retinal vessel segmentation algorithms [25,26].

We have shown that dense segmentation of CNP regions is a challenging task for both human graders and deep learning-based models. The performance reached by the fully convolutional network was similar to that obtained by human grader agreement. That behaviour has been confirmed when investigating the distribution of CNP in the concentric rings. Intergrader agreement has shown second grader had identified more regions as non-perfused, annotating 36.33% (12.93) of the gradable retina, compared to grader 1 annotation of 25.63% (12.81). Nonetheless, as reflected by recall value, some CNP regions remained unidentified by second grader, which confirmed the differences cannot simply be explained by a less strict threshold in CNP identification and demonstrate the variability and complexity of the task.

We have introduced a second stage in the automated methodology, that has demonstrated the potential of an automatic grid-based assessment to describe retinal CNP topology. The automated methodology, with an averaged kappa value of 0.55, demonstrates higher agreement than the second grader, reaching 0.43. ICC values on the numbers of perfused and nonperfused segments confirm the same trend, though showing much higher agreement in both cases. Furthermore, similar values of the ICC measure have been shown when independently assessed within the different rings. This suggests the strength of the automated algorithm, despite contrast and illumination challenges in different areas of the retina.

Cohen’s kappa values have proved to be a very strict measure, due to its consideration of agreement by chance. When kappa is measured independently for each image, agreement by chance is computed from that specific image. Similar annotations on mostly perfused (or non-perfused) retinas can result in low kappa values, because of the high agreement by chance. That case is illustrated by the bottom example in Figure 5, in which similar kappa values are achieved by the automated system and grader 1, despite important annotating differences.

The challenges in quantifying CNP on UWF-FA of the retina are as follows. Firstly, although UWF captures 200 degrees of the retina, only about 100 degrees of the retina is gradable for CNP, because of artefacts such as eyelashes obscuring the view, especially in the superior and inferior retina; the peripheral retina is normally non-vascularised in some areas in some patients and it is challenging to distinguish normal retinal non-perfusion from pathology. Therefore, we need to first identify the boundary of the gradable retina (region of interest). This boundary may not be the same in consecutive visits, so it is more pragmatic to only concentrate in changes in the posterior retina. Posterior retinal CNP is also a marker of increasing disease severity [27]. In order to ensure that, we measure the same area in consecutive visits, we used the validated concentric grid and named each sector or zone in the gradable retina to ensure we only compare the same gradable retina in both visits. 

Secondly, the quality of fundus images may be influenced by eye movement, non-alignment or poor focus of the camera, small pupil or uneven illumination. This requires image pre-processing. Deep learning on pre-processed images introduces challenges in translation to clinical practice. Moreover, there are significant variations in gradations in the ground glass appearance of CNP, so, even in gradable zones, there is a tendency to ignore small areas of CNP that are difficult to define manually. This study is an evidence of the low intra-grader and intergrader agreement. Here, we chose to train the deep learning on the images, where several subtle areas of CNP were missed to resemble as closely as possible to clinical practice. One could argue that the algorithm should be developed on the images in which all tiny areas of CNP were identified by the human grader, but we believe this will possibly result in the over fitting of the algorithm.

Whilst a rough global estimate of CNP was sufficient previously when no treatment was available, novel therapies are being evaluated to prevent progression or reverse CNP, and so a more accurate measure of CNP is required. This study shows that the agreement between deep learning algorithm and human grader is similar to human grader agreement, highlighting the potential use of this algorithm as an outcome measure in clinical trials and a decision tool in clinical practice.

## Figures and Tables

**Table 1:** Comparative of grid assessment of CNP (Grid1: manual grid assessment by grader 1, Grader 2: manual grid assessment by grader 2, AI expert: automatic assessment of AI expert dense segmentation, Grader1: automatic assessment of manual dense segmentation by grader 1, Grader 1: automatic assessment of the dense segmentation by grader 1, Grader 2: automatic assessment of the dense segmentation by grader 2, SE: standard error, CI: confidence interval).

CNP Descriptors	N. Images	Kappa (SE)	ICC Perfused (95% CI)	ICC Non-Perfused (95% CI)
Grid1 vs. Automated	75	0.55 (0.03)	0.88 (0.77, 0.93)	0.86 (0.73, 0.92)
Grader1 vs. Automated	75	0.65 (0.04)	0.90 (0.85, 0.94)	0.88 (0.81, 0.92)
Grid1 vs. Grid2	75	0.43 (0.03)	0.70 (0.48, 0.83)	0.71 (0.48, 0.83)
Grid1 vs. Grader1.	75	0.56 (0.03)	0.85 (0.54, 0.94)	0.82 (0.48, 0.92)
Grid2 vs. Grader2	20	0.44 (0.03)	0.78 (0.52, 0.91)	0.79 (0.54, 0.91)

**Table 2:** Number of perfused and non-perfused segments in each ring and ICC values (SD: standard deviation, CI: confidence interval).

	Grid1 (SD)		Automated (SD)		ICC (95% CI)	
	Perfused	Non-Perfused	Perfused	Non-Perfused	Perfused	Non-Perfused
M1	10.39 (2.51)	1.55 (2.52)	10.28 (2.53)	1.65 (2.54)	0.83 (0.74, 0.89)	0.83 (0.74, 0.89)
R1	6.43 (2.95)	3.64 (2.71)	7.37 (3.04)	2.69 (0.80)	0.84 (0.62, 0.92)	0.81 (0.57, 0.90)
R2	2.05 (2.15)	2.65 (2.12)	2.51 (2.19)	2.2 (2.0)	0.86 (0.76, 0.92)	0.85 (0.74, 0.91)
R3	0.49 (1.12)	0.95 (1.23)	0.68 (1.24)	0.76 (1.15)	0.83 (0.74, 0.89)	0.84 (0.75, 0.89)
R4	0.06 (0.34)	0.27 (0.70)	0.16 (0.69)	0.17 (0.47)	0.67 (0.53, 0.78)	0.71 (0.58, 0.81)
R5	0.03 (0.23)	0.07 (0.30)	0.01 (0.11)	0.08 (0.39)	0.80 (0.70, 0.87)	0.94 (0.92, 0.97)
